# Supplementary material for: Perceiving animacy in ‘identical’ images
Source: eLife. 2026 Aug 26;15:RP111179. doi: 10.7554/eLife.111179 (PMC13516682; doi:10.7554/eLife.111179)
Supplement: Peer review history

# Editor report: eLife Assessment

**DOI:** 10.7554/eLife.111179.3.sa0

**Authors:** Xilin Zhang

**Affiliations:** Key Laboratory of Brain, Cognition and Education Sciences, Ministry of Education, South China Normal University China

**Keywords:** Convincing, Important

This **important** study uses an elegant visual-anagram approach to test whether perceived animacy shapes visual working memory and guides visual attention while tightly controlling for lower- and mid-level image properties. The evidence is **convincing** and provides a rigorous demonstration that perceived animacy influences visual cognition beyond the contribution of its typical visual correlates. The findings will be of broad interest to researchers studying high-level vision, attention, and working memory.

# Referee report: Reviewer #1 (Public review)

**DOI:** 10.7554/eLife.111179.3.sa1

[Editors' note: this version has been assessed by the Reviewing Editor without further input from the original reviewers. The authors have addressed the comments raised in the previous round of review.]

This is a very cool paper that casts light on a persistent problem in the psychology and philosophy of visual representation: is there high-level perception? Every vision scientist agrees that low-level features such as shape, color, texture, motion and spatial frequency are represented in visual perception, but there is a great deal of controversy about the representation of high-level properties such as causation, faces, agency and animacy. Animacy is especially problematic because there are large differences in line curvature between stimuli that represent animate and inanimate items.

This article uses a novel approach-visual "anagrams" that are exactly the same image, except one is rotated 90 degrees relative to the other. They found persistent differences in visual processing between animate and inanimate stimuli. (Of course, the stimuli aren't animate-they represent animate items.). For example, there were processing differences between changes between animate and inanimate items (rabbit to boot) that were not present in rabbit to dog. They also showed such differences in two kinds of visual search tasks.

Of course, there are feature differences that exploit orientation. A classic example is the difference between a square and a diamond that is produced from the square by rotating it 45 degrees.

They addressed an aspect of this challenge having to do with some features using silhouettes. There was no search advantage for silhouetted stimuli.

# Referee report: Reviewer #2 (Public review)

**DOI:** 10.7554/eLife.111179.3.sa2

Summary:

The authors present a creative approach using visual anagrams matched on low-level image statistics to isolate animacy from low-level visual features and report consistent effects of animacy on visual working memory and attention.

Strengths:

(1) An important methodological advance in controlling low-level confounds that have historically complicated the study of animacy.

(2) The converging effects across multiple experiments, together with the pre-registered design, strengthen the reliability of the reported findings.

# Referee report: Reviewer #3 (Public review)

**DOI:** 10.7554/eLife.111179.3.sa3

This study makes clever use of generative AI to create stimuli that are pixel-for-pixel identical but which have radically different meanings depending on their orientation, to investigate the perception of animacy while retaining control over low-level image features (so-called 'anagram' stimuli).

The authors present seven elegantly designed experiments in a commendably compact format.

Experiments 1 and 2 involved a working memory paradigm in which participants had to spot which of five objects in an array changed after a pause. Importantly, the changed object was an anagram stimulus that in one orientation matched the animacy/inanimacy of the changed object, and in the other orientation was the opposite (e.g., a rabbit is replaced by either a dog or a boot, where the dog and boot stimuli are actually identical, just rotated by 90 degrees). They found a difference in accuracy depending on whether the animacy of the objects matched.

Experiments 3 and 4 used a visual search task in which the participants had to localize the target, and the distractors were anagrams that either matched the target in terms of animacy or did not. There was a significant cost in terms of response time when the animacy of the target was the same as that of the distractors. Experiments 5 and 6 also used a similar visual search design, except that the task was to determine if the target was present or absent from the display, and the distractors again either matched or differed from the target in terms of animacy. Again, the authors found slower responses when the distractor arrays matched the animacy of the target than when they differed.

An obvious potential concern about the studies is addressed by Experiment 7. It is unclear if the observed effects are related to the specific orientations of the target and distractor stimuli selected in each condition. For example, it could be that all the animate versions of the anagrams involved tall and skinny shapes, while all the inanimate versions involved wide and short objects, due to the 90-degree rotational difference between the two versions of the stimuli. To control for this, the authors repeated the visual search experiment but with convex-hull silhouettes of each of the stimuli. In other words, all targets and distractors from each trial were replaced by a black splotch with approximately the same overall outline (envelope) as the corresponding stimulus. Importantly, in contrast to the anagram stimuli, the silhouettes had had no meaningful semantic interpretation, and their animacy did not change depending on their orientation.

# Author comment: Author response

**DOI:** 10.7554/eLife.111179.3.sa4

**Authors:** Tal Boger, Chaz Firestone

**Affiliations:** Johns Hopkins University Baltimore United States; Johns Hopkins University Baltimore United States

The following is the authors’ response to the original reviews.

**eLife Assessment**

This valuable study uses an elegant visual-anagram approach to test whether perceived animacy structures visual working memory and attention while controlling for many low-level image properties. The evidence is solid, with converging results across seven preregistered experiments, but the central claim that animacy itself is represented independently of visual features should be tempered, as residual mid-level configural cues, ensemble or category structure, and broader semantic differences may also contribute to the effects. The work will be of interest to researchers studying high-level visual representation, attention, and working memory.

We thank the Editors and Reviewers for this careful and informed assessment. We appreciate that every Reviewer found our approach to be elegant, our findings to be solid, and our question to be of broad interest. We respond to each Reviewer’s specific comments in more detail below; but we thought to summarize some of the highlights - especially the specific comments that come up in this Assessment - here.

(1) The Reviewers make the insightful point that, even if our stimuli effectively control for many low-level features, there may be other high-level features that explain performance in our experiments (R2: “Although the anagram paradigm effectively controls low-level visual features […] these stimuli differ not only in animacy but also along other semantic dimensions such as natural versus manmade categories.”). We are happy to embrace this possibility. If our results were explained by high-level visual representation of the natural vs. manmade distinction, rather than the animate vs. inanimate distinction, this would still be an appeal to a (not altogether unrelated) high-level property being represented independently from its lower-level features, which was the primary motivation for our study. We framed our work specifically around animacy given the persistent debates regarding perceived animacy, as well as the fact that our stimuli do quite saliently vary along that dimension; but we are certainly open to other nearby high-level categories being at play. We also think this is an empirical question that could be tested in future work. For example, objects like rocks and lakes are natural but inanimate. If they behave more like dogs than like boots in our paradigms, then Reviewer #2 may be right that naturalness was the relevant property all along; but if they behave more like boots than like dogs, then perhaps it really was animacy doing the work. We now discuss this explicitly in our paper, and we appreciate the opportunity to not only clarify our claims but also spur discussion for future work.

(2) Multiple Reviewers raise the question of whether semantic factors that go beyond the images themselves may be driving our effects. Reviewer #3 raises a particularly interesting question along these lines: “if all the stimuli in the experiments were replaced with the verbal names of the depicted objects instead of pictures, would we expect different results?” We have now taken this question quite literally and run this experiment exactly as described. Of course, much research already explores cognitive processing of animate/inanimate words, finding (for example) stronger memory for animate objects than inanimate ones (e.g., Nairne et al., 2013; Nairne et al., 2017). However, such tasks do not invoke effects of visual processing, whereas the question at issue here is specifically whether the visual system prioritizes animacy independent of its lower-level features. To this end, we conducted a new, pre-registered experiment (now Experiment 8) where participants search for animate/inanimate words on some trials, and animate/inanimate pictures on others. Given the nature of visual search tasks, we should expect to find no search advantage for words (as their meanings are not processed in vision per se) — and we should also expect to replicate (once again) our search advantage for pictures. This is exactly what we found. In other words, linguistic stimuli alone failed to produce the effect, while anagrams did produce the effect. We believe this rules out the strongest form of the semantic labeling account.

(3) Finally, Reviewers #2 and #4 raise some concerns regarding residual mid-level features such as configural shape and ensemble statistics, which lie somewhere between animacy itself and more basic properties like contrast or spatial frequency. In our paper, we now clarify each of these concerns in greater detail. In short: We think that our stimuli and experiments indeed control for these residual cues. For example, rotating an image preserves its configural shape; and, as we argue below, the specific ensemble statistics argument fails to get off the ground without appeal to animacy itself.

**Public Reviews:**

**Reviewer #1 (Public review):**

Summary:

Evidence for visual representation of animacy.

Strengths:

This is a very cool paper that casts light on a persistent problem in the psychology and philosophy of visual representation: is there high-level perception? Every vision scientist agrees that low-level features such as shape, color, texture, motion and spatial frequency are represented in visual perception, but there is a great deal of controversy about the representation of high-level properties such as causation, faces, agency and animacy. Animacy is especially problematic because there are large differences in line curvature between stimuli that represent animate and inanimate items.

This article uses a novel approach-visual "anagrams" that are exactly the same image, except one is rotated 90 degrees relative to the other. They found persistent differences in visual processing between animate and inanimate stimuli. (Of course, the stimuli aren't animate-they represent animate items). For example, there were processing differences between changes between animate and inanimate items (rabbit to boot) that were not present in rabbit to dog. They also showed such differences in two kinds of visual search tasks.

Of course, there are feature differences that exploit orientation. A classic example is the difference between a square and a diamond that is produced from the square by rotating it 45 degrees.

They addressed an aspect of this challenge having to do with some features using silhouettes. There was no search advantage for silhouetted stimuli.

Weaknesses:

I thought this was an excellent submission. I have two suggestions for revision:

We are glad to hear this Reviewer recognizes the broad challenge we are tackling in this work (separating high-level from low-level visual features) and found our submission to be “excellent”.

(1) I thought that experiment 7 should have been described in more detail, with the upshot explained better. What exactly do the authors take it to show?

Sorry for the lack of clarity here. We think the Reviewer actually gets this right earlier in their review; many feature differences exploit orientation, and our silhouettes control (Experiment 7) shows that those differences alone fail to explain our effects. For example, one might worry that our search effects merely reflect oddities in the aspect ratio or center of mass of the images. Converting the anagrams into silhouettes preserves these features. Thus, the fact that we found no search advantage with silhouettes suggests that these features on their own fail to produce the relevant effects; put the other way around, the effects we observed earlier must go beyond those features. We now discuss this in greater depth in our paper.

(2) There should be a candid discussion of what the loose ends are and how they might be addressed. It would be good to have some examples like the square/diamond case with some indication of what would address such challenges.

We agree with this, though we are somewhat limited by the space constraints of the Short Report format. A primary loose end we see is the possibility that high-level properties other than animacy explain our results (as raised by other Reviewers). We have added some discussion of this possibility to the paper.

We would like to thank this Reviewer for their thoughtful feedback.

**Reviewer #2 (Public review):**

Summary:

The authors present a creative approach using visual anagrams matched on low-level image statistics to isolate animacy from low-level visual features and report consistent effects of animacy on visual working memory and attention. While this is a thoughtful design and is well executed across seven pre-registered experiments, it remains unclear whether the reported effect is truly driven by animacy, as opposed to broader differences in ensemble statistics or semantic structure across the "mixed animacy" versus "uniform animacy" conditions. As such, the interpretation of a "pure" animacy effect may be overstated.

Strengths:

(1) An important methodological advance in controlling low-level confounds that have historically complicated the study of animacy.

(2) The converging effects across multiple experiments, together with the pre-registered design, strengthen the reliability of the reported findings.

We are glad to hear this Reviewer found our work to be “creative” and believes it offers an “important methodological advance”.

Weaknesses:

(1) Specificity of the animacy effect vs. category-level ensemble structure

The central claim is that animacy itself drives the observed effects. However, the key manipulation ("mixed animacy" versus "uniform animacy") also introduces differences in category-level ensemble structure. For example, in Experiments 1-2, cross-category change detection (e.g., dog to chair) may be easier not because of animacy per se, but because of a change in overall ensemble statistics (Brady & Alvarez, 2011, 2015). In addition, since each display contains five objects (two in one category and three in the other category), cross-category changes may also alter category balance in a way that further facilitates detection. In contrast, within-category changes preserve both ensemble structure and category composition, making them more difficult to detect.

Brady, T. F., & Alvarez, G. A. (2011). Hierarchical encoding in visual working memory: Ensemble statistics bias memory for individual items. Psychological Science.

Brady, T. F., & Alvarez, G. A. (2015). Contextual effects in visual working memory reveal hierarchically structured memory representations. Journal of Vision.

We appreciate the opportunity to clarify our claims and the support for them. Our claim is indeed that animacy (or a closely related high-level property; see below) drives our effects, over and above its lower-level correlates — i.e., that the explanation for differences in change detection or search across conditions will invoke a high-level property of the images. As we understand the Reviewer’s concern(s), they either (a) are already addressed by our novel methodology, or (b) would still fall perfectly in line with our claim as stated above.

Consider the Reviewer’s concern that cross-category change detection “may be easier not because of animacy per se, but because of a change in overall ensemble statistics”. Which ensemble statistics change across categories in our stimulus set? Take as an example the case depicted in our figure, where a rabbit changes into either a dog (within-category) or a boot (cross-category). The dog and the boot are the very same image, just rotated; thus, they have the same luminance, curvature, area, spatial frequency, and so on. So if the change from rabbit to dog changes the array’s ensemble statistics with respect to any of those properties, it does so in the very same way as the change from rabbit to boot — and yet detection is still better for rabbit → boot than for rabbit → dog. Indeed, for nearly any ensemble statistic, the difference between the rabbit-display and the dog-display will be identical to the difference between the rabbit-display and the boot-display. To engage with the specific cases discussed in the two cited papers (Brady & Alvarez, 2011, 2015): The dog and the boot are the same size (because they are the same image), so average size is identical (just as average luminance, curvature, area, and spatial frequency are identical). And the very few properties left over (e.g., aspect-ratio) are addressed by later experiments.

To be clear: We are not saying that there are no differences in ensemble statistics between the rabbit-display and the dog-display; across those displays, we replace one image with a different image, so there are likely all kinds of corresponding differences in ensemble statistics. The key question is whether that change in ensemble statistics differs across trial types in ways that might explain our effect - i.e., whether there is any difference between the rabbit-display and dog-display that is not also present between the rabbit-display and the boot-display. We don’t see how the answer could be yes, at least with respect to the statistics typically considered. A similar logic applies to the search tasks, with the silhouette control (Experiment 7) providing especially strong evidence that certain ensemble statistics or lower-level features cannot explain our effect.

Now, it’s possible the Reviewer is referring to properties other than the low-/mid-level properties we mention above. Perhaps, for example, many animate stimuli on a display at one time have a striking collective appearance (all these animals are looking at me!) that lots of inanimate stimuli do not (this might be related to the Reviewer’s concern about “category balance”). But as we see it, this explanation just invokes animacy all over again, and so is the sort of explanation we would embrace.

We now say more about this concern in the paper to be as clear as possible about our claims.

(2) Limited stimulus set and potential learning effects

The relatively small stimulus set (six anagram pairs) and repeated exposure raise the possibility of learning or familiarity effects. Does performance change over time? e.g., are there meaningful differences between early and late trials (e.g., first 10% vs. last 10%)? If such differences are present, they could suggest the development of task-specific strategies or increased efficiency with repeated exposure, rather than stable effects driven by the experimental manipulation itself.

This is an interesting question, and we recognize this analysis absent from our initial submission. To be fair, stimulus sets of this size are not unusual in change-detection and search tasks, which often involve red, green, and blue squares repeated over the course of several hundred trials. Still, we certainly take the Reviewer’s point here and also embrace their analytical approach to addressing it. We’ve now run the “familiarity effects” analyses the Reviewer suggests (as well as some they did not suggest). The top-level headline is that learning or familiarity effects cannot explain our results, and if anything most of these analyses not only fail to support this alternative account but actively point against it. Below are more details.

First, we worry that the Reviewer’s concern about “the development of task-specific strategies … rather than stable effects driven by the experimental manipulation itself” isn’t actually addressed by the suggested analysis of comparing the last 10% of trials to the first 10%. One reason for this is simply that it’s possible that both mechanisms are at play - i.e., that there is a baseline difference even without any familiarity that is then enhanced by some learning mechanism. (There are other issues as well: For example, one might imagine that participants get quite good at the task during the middle 80% of trials, but then get fatigued at the end. If this were true, then comparing the first 10% to the last 10% of trials could make it seem like there is no learning or familiarity, even if there were such effects. And on top of all this there is just the issue of statistical power, since far fewer trials go into these analyses than into our primary, pre-registered analyses). Nevertheless, we ran the Reviewer’s proposed analyses (using the first and last 10 trials of each type, which offers the best chance to find the pattern the Reviewer is concerned about). If anything, this analysis points in the opposite direction to the Reviewer’s prediction: 4/6 experiments (Experiments 1, 3, 4, and 5) revealed numerically weaker effects at the end of the task than the start, while only 2/6 experiments (Experiments 2 and 6) revealed numerically stronger effects at the end of the task than the start. Moreover, most of these results were non-significant, with only one marginal result (Experiment 5, *p*< = 0.08) and one significant result (Experiment 2, *p* = 0.01), and this is before any correction for multiple comparisons, which would make all of these results non-significant. So even though our account could easily accommodate learning effects, it’s not clear that they even exist here in any consistent or reliable way.

Second, however, we think a more informative way to answer the Reviewer’s question is to ask not about learning over the course of the experiment but rather whether the key effects arise very early in the task. If they do, then any learning effects arising later couldn’t fully account for our results. Now, again, these tests are underpowered and only exploratory (to do this analysis properly, we would want to run entirely new experiments designed for this purpose), but we in fact did find evidence that our key effects arise early. In 5/6 experiments (Experiments 1, 3, 4, 5, and 6), the key effect was significantly (or in one case marginally) present even at the beginning of the experiment (Experiment 1, *p* = 0.07; Experiment 3, *p* = 0.01; Experiment 4, *p* < 0.001; Experiment 5, *p* < 0.001; Experiment 6, *p* < 0.01), and most of these results would survive correction for multiple comparisons. (In only one experiment, Experiment 2, was there a numerical disadvantage, but it was not significant; *p* = 0.34.) So even though our experiments were not designed or powered for this purpose, they do seem to suggest that the effects arise even without much familiarity at all.

All told, we think these analyses suggest quite strongly that learning alone fails to explain our key effects. There is no evidence that the effects in general are stronger at the end of the experiment than the beginning (if anything it is the opposite); and there is evidence that most of the effects we investigated can be detected even very early in the experimental sessions. We have added discussion of these new analyses to our manuscript.

(3) Role of semantics

Although the anagram paradigm effectively controls low-level visual features, it still relies on high-level semantics (e.g., "dog" vs. "boot"). These stimuli differ not only in animacy but also along other semantic dimensions such as natural versus manmade categories. From a semantic standpoint, it remains unclear whether the observed effects can be uniquely attributed to animacy or whether they reflect broader conceptual distinctions.

We agree with the Reviewer here. While we feel comfortable interpreting our effects in terms of a high-level property like animacy as opposed to a lower-level property like curvature, it remains possible that the observed effects reflect some other, closely related high-level distinction (like natural vs. manmade). Our primary concern was to tease apart high-level properties from low-level features, which the Reviewer’s question does not threaten — if attention and memory are sensitive to the natural/artificial distinction, that’s interesting too, and a near neighbor of our actual claim. Still, we agree that this could be addressed, and we even see it as an empirical question testable in future work. Perhaps the most relevant departures between animate/inanimate and natural/manmade include objects like clouds, plants, and rocks — objects that are natural but not “animate” in the sense often used in this literature. If something like our paradigm revealed that rocks behave more like dogs than like boots, that would suggest that naturalness, rather than animacy, was driving the effects; but if rocks behave more like boots than like dogs, that would point to animacy even more strongly. We remain open-minded about this possibility, but it would of course require multiple new experiments with a brand new stimulus set and so goes beyond the present contribution. In any case, we have added a discussion of this issue to the paper and have adjusted our claims accordingly.

**Reviewer #3 (Public review):**

Summary:

This study makes clever use of generative AI to create stimuli that are pixel-for-pixel identical but which have radically different meanings depending on their orientation, to investigate the perception of animacy while retaining control over low-level image features (so-called 'anagram' stimuli).

The authors present seven elegantly designed experiments in a commendably compact format.

Experiments 1 and 2 involved a working memory paradigm in which participants had to spot which of five objects in an array changed after a pause. Importantly, the changed object was an anagram stimulus that in one orientation matched the animacy/inanimacy of the changed object, and in the other orientation was the opposite (e.g., a rabbit is replaced by either a dog or a boot, where the dog and boot stimuli are actually identical, just rotated by 90 degrees). They found a difference in accuracy depending on whether the animacy of the objects matched.

Experiments 3 and 4 used a visual search task in which the participants had to localize the target, and the distractors were anagrams that either matched the target in terms of animacy or did not. There was a significant cost in terms of response time when the animacy of the target was the same as that of the distractors. Experiments 5 and 6 also used a similar visual search design, except that the task was to determine if the target was present or absent from the display, and the distractors again either matched or differed from the target in terms of animacy. Again, the authors found slower responses when the distractor arrays matched the animacy of the target than when they differed.

An obvious potential concern about the studies is addressed by Experiment 7. It is unclear if the observed effects are related to the specific orientations of the target and distractor stimuli selected in each condition. For example, it could be that all the animate versions of the anagrams involved tall and skinny shapes, while all the inanimate versions involved wide and short objects, due to the 90-degree rotational difference between the two versions of the stimuli. To control for this, the authors repeated the visual search experiment but with convex-hull silhouettes of each of the stimuli. In other words, all targets and distractors from each trial were replaced by a black splotch with approximately the same overall outline (envelope) as the corresponding stimulus. Importantly, in contrast to the anagram stimuli, the silhouettes had had no meaningful semantic interpretation, and their animacy did not change depending on their orientation.

Strengths:

The main strength is the elegant use of stimuli that control almost perfectly for low-level image features.

Thank you for this kind feedback. This summary perfectly captures both our empirical contribution and the claims we are making.

Weaknesses:

My only real concern about the study is whether the findings truly provide evidence for a high-level visual representation of animacy independent of the low-level stimulus characteristics, or whether, instead, the effects are essentially semantic priming, which is independent of visual processing per se. For example, if all the stimuli in the experiments were replaced with the verbal names of the depicted objects instead of pictures, would we expect different results? Words can also access semantic representations of the animacy of objects, and also don't suffer from low-level visual confounds. It would be helpful to add a discussion of this possibility to the article.

Wow, we love this question! And so we’ve now conducted exactly the experiment the Reviewer suggests here. In a new pre-registered study (Experiment 8), we presented participants with a present/absent search task (as in Experiments 5–7). One half of trials consisted of the anagram stimuli (such that we could, once again, replicate the mixed-animacy search advantage); but the other half of trials consisted of the words describing the anagrams (e.g., “dog”, “boot”, “sheep”, “car”, etc.). The experiment worked beautifully: We found no effect with the words, but replicated the search advantage with the pictures — and also found a significant difference between the effects elicited by the two stimulus types.

We agree with the Reviewer that this now rules out the possibility that semantic representations alone explain these visual effects. Thank you!

**Reviewer #4 (Public review):**

In this article, the authors investigate whether perceived animacy influences visual processing independently of lower-level visual features by using "visual anagrams." Across seven experiments, they test whether animacy, isolated from many lower-level visual properties, structures visual working memory and guides visual attention. The central claim is that the visual system may represent animacy itself, rather than animacy emerging solely from associations among low-level visual properties.

I find this investigation compelling. The experiments described provide strong control over several lower-level visual features, including curvature, texture, and related image properties. However, the visual anagrams are not pixelwise-identical across orientations. Because the images are rotated, the retinal configuration of pixels and the spatial organization of some low- to mid-level shape features also change. As a result, the configural arrangement of mid-level visual features may still contribute to perceived animacy.

We are glad to hear the Reviewer finds our investigation “compelling”.

I encourage the authors to discuss how independent perceived animacy is in this context from the contribution of mid-level visual features, such as configural shape cues that are diagnostic of animacy. This distinction would help sharpen the interpretation of the results and more precisely define the level of visual representation isolated by the visual-anagram approach.

This is a helpful point, and it also echoes a sentiment expressed by Reviewer #2. While configural shape is diagnostic of animacy writ large, it can’t account for our observed effects here because rotating an image does not vary its configural shape. We now mention this in our work, and we agree that it helps sharpen the interpretation of our studies.

Additionally, previous studies have argued that low- and mid-level curvilinear features may contribute to animate/inanimate categorization, and may in some cases be sufficient to support such distinctions (e.g., PMID: 33798259; PMID: 28654965). I encourage the authors to clarify how these previous findings on curvilinearity and rectilinearity fit with the overarching claim of the current study, namely that the visual system may represent animacy itself rather than animacy emerging solely from associations among lower-level visual properties.

Yes, many studies from exactly that corner of the field actually motivated the present work, which is why we cited them in our submission. In a way, we are approaching this issue from the other side of the equation. Whereas the papers the Reviewer points to (along with many others) ask whether mid-level features (such as curvilinearity and rectilinearity) are sufficient to support perceived animacy, we ask whether these and other features are necessary to support perceived animacy. Prior work is relatively split on this issue, leaving the question wide open. We take our work to show that differences in curvature are not necessary for differences in perceived animacy, because our anagrams have identical curvature yet differ in animacy — and the visual system capitalizes on that difference. Put the other way around, representation of animacy can and does go beyond representation of its low- and mid-level correlates. Thank you!
